# Adherence to quarterly HIV prevention services and its impact on HIV incidence in men who have sex with men in West Africa (CohMSM ANRS 12324 – Expertise France)

**DOI:** 10.1186/s12889-021-10994-4

**Published:** 2021-05-22

**Authors:** Ter Tiero Elias Dah, Issifou Yaya, Luis Sagaon-Teyssier, Alou Coulibaly, Malan Jean-Baptiste Kouamé, Mawuényégan Kouamivi Agboyibor, Kader Maiga, Issa Traoré, Marion Mora, Paméla Palvadeau, Daniela Rojas-Castro, Fodié Diallo, Ephrem Mensah, Camille Anoma, Bintou Dembélé Keita, Bruno Spire, Christian Laurent, on behalf of the CohMSM Study Group, Christian Laurent, Issifou Yaya, Sayouba Ouedraogo, Bruno Granouillac, Laetitia Serrano, Martine Peeters, Clotilde Couderc, Bruno Spire, Luis Sagaon-Teyssier, Marion Mora, Gwenaëlle Maradan, Michel Bourrelly, Pierre-Julien Coulaud, Cyril Berenger, Daniela Rojas Castro, Adeline Bernier, Paméla Palvadeau, Bintou Dembélé Keita, Fodié Diallo, Alou Coulibaly, Kader Maïga, Drissa Camara, Mahamadou Diarra, Aly Ouologuem, Abdoul Aziz Keita, Oumar Cissé, Fodé Traoré, Bréhima Abdrahamane Ouary, Ibrahima Kanta, Camille Anoma, Malan Jean-Baptiste Kouame, Rachelle Kotchi, Niamkey Thomas Aka, Kpassou Julien Lokrou, Noufo Hamed Coulibaly, Jean Armel Ekessi Koffi, Frédéric Dibi N’guessan, Stéphane-Alain Babo Yoro, Adama Cissé, Ter Tiero Elias Dah, Issa Traoré, Camille Rajaonarivelo, Juste Rodrigue Touré, Joseph Ouedraogo, Christian Coulibaly, Mamadou Ouedraogo, Elisabeth Thio, Ousseni Ilboudo, Abdoulazziz Traoré, Honoré Comsiambo, Ephrem Mensah, Richard Mawuényégan Kouamivi Agboyibor, Anani Attisso, Anouwarsadat Kokouba, Aléda Mawuli Badjassim, Kouakou Kokouvi Selom Agbomadji, Messan Attiogbe, Kossi Jeff Yaka, Agbégnigan Lorette Ekon, Julien Bimba, Claver Anoumou Yaotsè Dagnra

**Affiliations:** 1 Association African Solidarité, Ouagadougou, Burkina Faso; 2 TransVIHMI, Univ Montpellier, Inserm, IRD, Montpellier, France; 3 Institut National de Santé Publique, Centre Muraz, Bobo-Dioulasso, Burkina Faso; 4 SESSTIM, Aix Marseille Univ, Inserm, IRD, Marseille, France; 5 ARCAD-SIDA, Bamako, Mali; 6 Espace Confiance, Abidjan, Côte d’Ivoire; 7 Espoir Vie Togo, Lomé, Togo; 8 Coalition Internationale Sida, Pantin, France

**Keywords:** HIV, Prevention, Incidence, MSM, Africa

## Abstract

**Background:**

Access to tailored HIV prevention services remains limited for West African MSM. We assessed adherence to quarterly HIV prevention services and its impact on HIV incidence in MSM followed up in four cities in Burkina Faso, Côte d’Ivoire, Mali, and Togo.

**Methods:**

We performed a prospective cohort study between 2015 and 2018. HIV-negative MSM aged over 18 benefited from quarterly medical visits which included a clinical examination, HIV testing, screening and treatment for other sexually transmitted infections, peer-led counselling and support, and the provision of condoms and lubricants. Determinants of adherence to quarterly follow-up visits and incident HIV infections were identified using generalized estimating equation models and Cox proportional hazard models, respectively.

**Results:**

618 MSM were followed up for a median time of 20.0 months (interquartile range 15.2–26.3). Overall adherence to quarterly follow-up visits was 76.5% (95% confidence interval [CI] 75.1–77.8), ranging from 66.8% in Abidjan to 87.3% in Lomé (*p* < 0.001). 78 incident HIV infections occurred during a total follow-up time of 780.8 person-years, giving an overall incidence of 10.0 per 100 person-years (95% CI 8.0–12.5). Adherence to quarterly follow-up visits was not associated with the risk of incident HIV infection (adjusted hazard ratio 0.80, 95% CI 0.44–1.44, *p* = 0.545).

**Conclusions:**

Strengthening HIV prevention services among MSM in West Africa, including the use of PrEP, will be critical for controlling the epidemic, not only in this key population but also in the general population. Quarterly follow-up of MSM, which is essential for PrEP delivery, appears feasible.

**Trial registration:**

ClinicalTrials.gov, number NCT02626286 (December 10, 2015).

## Background

Men who have sex with men (MSM) are disproportionately affected by HIV in Africa and elsewhere [1–3]. In 2018, their risk of acquiring HIV was 22 times higher than that of all male adults. In Western and Central Africa, the HIV epidemic is concentrated in key populations (including MSM, sex workers, and people who inject drugs), which together with their sexual partners represented 64% of all new HIV infections in 2018 [4]. Furthermore, median HIV prevalence in 16 West African countries was 13.7% among MSM compared with 1.5% in the general population. In addition, HIV infection in African MSM contributes to the dynamic of the epidemic [5, 6] because 50–90% of MSM also have sex with women [7, 8].

Since the early 2010s, the World Health Organization (WHO) has recommended targeting MSM as part of the response to the HIV epidemic [9, 10]. More specifically, recommendations encourage healthcare decision-makers to promote routine HIV testing and counselling in this population. MSM are also encouraged to perform HIV retesting at least annually and every three months if they engage in high-risk sexual behaviours. Since 2014, the WHO has also recommended developing comprehensive prevention and care services which not only include the abovementioned HIV testing and counselling but also pre-exposure prophylaxis (PrEP), linkage and enrolment in care, HIV treatment and care, prevention and management of co-infections and other co-morbidities, as well as sexual and reproductive health interventions [11].

Risk factors for HIV acquisition in MSM include high per-act transmission probability of receptive anal sex, high rate of sexually transmitted infections (STI), high frequency of condomless anal sex, high number of male partners, and involvement in large sexual networks [2, 12, 13]. Moreover, MSM in Africa face social and legal barriers (i.e., homophobia, stigmatization, discrimination, violence, and penalization of same-sex relationships) that hinder their access to HIV prevention and care services [14, 15]. Unfortunately, HIV services tailored to their needs are limited and are mostly offered by community-based organizations [16, 17]. Furthermore, in general, HIV-negative MSM come to prevention services when they have a health need (e.g., STI) or for specific needs (e.g., condoms and lubricants).

The CohMSM study was designed to assess the feasibility and interest of implementing quarterly HIV prevention and care services in MSM in West Africa. In the present analysis, we assessed adherence to quarterly prevention services and its impact on HIV incidence in MSM followed up in four West African countries. Our study hypothesis was that high adherence to quarterly prevention services would be associated with lower HIV incidence.

## Methods

### Study design, setting and participants

A prospective cohort study was performed between June 2015 and January 2018 in Abidjan (Côte d’Ivoire), Bamako (Mali), Lomé (Togo), and Ouagadougou (Burkina Faso). MSM were enrolled and followed up in community-based clinics already providing MSM-specific prevention, care, and support (*Clinique de Confiance* in Abidjan, *Clinique des Halles* in Bamako, *Centre Lucia* in Lomé, and *Centre Oasis* in Ouagadougou). MSM were eligible if they were aged 18 or over, reported at least one episode of anal intercourse with another man in the previous three months, and were HIV negative (status confirmed at study enrolment). At enrolment and during the quarterly follow-up visits, participants benefited from a clinical examination, HIV testing, screening and treatment for other STI, personalized peer-led counselling and support, and the provision of condoms and lubricants. PrEP was not available. MSM who seroconverted during follow-up were invited to initiate antiretroviral therapy (ART) immediately. Participants could also attend the clinics at any time according to their needs. All services were free of charge. Participants were compensated 3000 Francs CFA (approximately US$5) for transport costs for each scheduled follow-up visit. Socio-demographic and behavioural data were collected at enrolment and every six months thereafter using a standardized face-to-face questionnaire administered by trained research assistants. Finally, with their consent, peer-educators could contact the participants by phone if they were 15 days late for their scheduled visits. The study was discontinued when PrEP was added to the CohMSM cohort. Specifically, the study was performed from June 2015 to January 2018 in Bamako, from October 2015 to January 2018 in Abidjan, from February 2016 to November 2017 in Ouagadougou, and from June 2016 to November 2017 in Lomé.

### Laboratory procedures

Screening for HIV was performed according to national algorithms [18–21]. All four study cities first used the Determine HIV 1/2 assay (Abbott Laboratories, Chiba, Japan). Positive results were confirmed using the Bioline HIV-1/2 3.0 assay (SD, Gyeonggi-do, Republic of Korea) in Abidjan, Bamako, and Ouagadougou, or the First Response HIV-1/2 assay (Premier Medical Corporation, Mumbai, India) in Lomé. Samples with discordant results were tested a third time using the HIV 1/2 Stat-Pak assay (Chembio Diagnostics, New York, USA) in Abidjan, the First Response HIV-1/2 assay (Premier Medical Corporation, Mumbai, India) in Bamako, the Inno-Lia HIV I/II Score assay (Fujirebio, Zwijnaarde, Belgium) in Lomé, or a Western Blot assay in Ouagadougou.

### Outcomes

#### Adherence to quarterly follow-up visits

Adherence to quarterly follow-up visits was defined as the proportion of scheduled visits which MSM actually attended (i.e., the number of attended visits divided by the number of scheduled visits). A visit was considered attended when it was carried out at the scheduled date plus or minus 45 days [22]. The period of participation for each participant (hereafter called “observation time”) ended at study discontinuation, first HIV positive screening test, or death.

#### HIV incidence

Incident HIV infection was defined as a HIV infection detected during follow-up. The date of HIV infection was estimated as the midpoint between the date of the last negative screening test and the date of the first positive one. HIV incidence was calculated per 100 person-years. Follow-up time was calculated from enrolment to HIV infection or the last participant’s HIV screening test.

### Explanatory variables

Potential explanatory variables considered in this analysis included: i) socio-demographic characteristics: city, age, education (primary school or higher versus never attended school or koranic school), marital status (married or in free union versus single/divorced/separated/widowed), ii) psychosocial characteristics: self-defined sexual orientation (homosexual or gay/heterosexual/bisexual versus transsexual/transgender), self-identified gender (much more a woman/both a man and a woman/neither man nor woman versus a man or a boy), sexual attraction (to men and women/to women versus to men), received psychological support (yes versus no); iii) sexual behaviours in the previous 6 months: condom use during insertive anal sex (inconsistent, no insertive anal sex versus consistent), condom use during receptive anal sex (inconsistent, no receptive anal sex versus consistent), received payment (whether financial or other) for transactional sex with male partners (sometimes, always versus never), provided payment (whether financial or other) for transactional sex with male partners (sometimes, always versus never), number of male sexual partners (1–5 versus ≥ 6); iv) clinical data: history of HIV screening (no versus yes), STI (other than HIV) symptoms (yes versus no).

### Statistical analysis

Adherence to quarterly follow-up visits was calculated overall and according to each study city. The 95% confidence intervals (CI) of adherence were computed using the binomial method. The evolution of adherence during follow-up was assessed using the χ^2^ test for trend. Determinants of adherence to quarterly follow-up visits were identified using generalized estimating equation models which provide population-averaged estimates while controlling for the correlation of repeated measures for the same individual. Independent variables associated with adherence with a *p*-value < 0.20 in univariate analyses were selected for the complete multivariate model. A backward elimination procedure based on the quasi-likelihood Akaike’s information criterion was used to determine the final multivariate model.

Cumulative hazards of incident HIV infections were estimated by Kaplan-Meier survival curves and compared between the study cities using the log-rank test. Given the fact that the proportional hazards hypothesis using the Schoenfeld residuals was verified for the most important covariates (i.e., adherence to quarterly follow-up visits and study city), the determinants of incident HIV infections were investigated using Cox regression models. Independent variables associated with incident HIV infections with a *p*-value < 0.20 in univariate analyses were specified in the complete multivariate model. A manual backward selection based on the log-likelihood method was used to determine the final multivariate model.

For the analyses of both outcomes, we used time-constant and time-dependent variables. The former were collected at enrolment and included the study city, age, educational level, marital status, and history of HIV screening. The latter included self-defined sexual orientation, self-identified gender, sexual attraction, condom use, sexual behaviours, psychological support, and STI (i.e., other than HIV) symptoms.

For all calculations, statistical significance was defined with a *p*-value < 0.05. All statistical analyses were performed using Stata software (version 15; Stata Corp LP, College Station, Texas). The CohMSM study is registered with ClinicalTrials.gov, number NCT02626286).

## Results

### Characteristics of participants

A total of 618 HIV-negative MSM were enrolled: 249 (40.3%) in Bamako, 133 (21.5%) in Abidjan, 121 (19.6%) in Ouagadougou, and 115 (18.6%) in Lomé. Their baseline characteristics are described in Table 1. Median age was 23.7 years (interquartile range [IQR] 21.2–27.0). Three hundred and thirty-six (54.5%) participants self-defined as bisexual, and 230 (37.3%) as homosexual/gay. A total of 354 (57.4%) participants self-identified as a man/boy, and 219 (43.6%) as both a man and a woman. The majority of participants (*n* = 319, 51.8%) were sexually attracted to men. With regard to STI risky behaviours in the previous six months, 185 (30.3%) participants reported inconsistent condom use during insertive anal sex, 206 (33.7%) reported inconsistent condom use during receptive anal sex, and 194 (38.8%) received payment (whether financial or other) for transactional sex with male partners. A large majority of participants (*n* = 532, 86.1%) had already been tested for HIV before study enrolment. Seventy-eight (12.6%) participants had at least one STI symptom (urethral or anal discharge, genital or anal ulceration, or condyloma) at enrolment.

**Table 1:** Baseline characteristics of the 618 MSM participants

	All (***N*** = 618)			Bamako (***N*** = 249)			Abidjan (***N*** = 133)			Ouagadougou (***N*** = 121)			Lomé (***N*** = 115)			
	N	n	(%)	N	n	(%)	N	n	(%)	N	n	(%)	N	n	(%)	***P***
**Age (years)**^a^	618	23.7	(21.2–27.0)	249	23.4	(21.1–26.3)	133	24.4	(22.1–27.7)	121	23.2	(20.9–26.6)	115	23.6	(20.3–27.3)	0.031
≤ 25		388	(62.8%)		166	(66.7%)		74	(55.6%)		82	(67.8%)		66	(57.4%)	0.065
**Educational level**	562			227			122			109			104			< 0.001
Never attended school/Koranic		19	(3.4%)		11	(5.4%)		5	(4.1%)		3	(2.7%)		0	–	
Primary school		72	(12.8%)		48	(20.5%)		6	(4.9%)		10	(9.2%)		7	(7.7%)	
Secondary school		250	(44.5%)		88	(36.6%)		44	(36.1%)		60	(55.1%)		58	(55.8%)	
University		221	(39.3%)		80	(37.5%)		67	(54.9%)		36	(33.0%)		38	(36.5%)	
**Marital status**	562			227			122			109			104			< 0.001
Married/free union		100	(17.8%)		20	(8.8%)		64	(52.4%)		9	(8.3%)		7	(6.8%)	
Single/divorced/separated/widowed		462	(82.2%)		207	(91.2%)		58	(47.6%)		100	(91.7%)		97	(92.2%)	
**Self-defined sexual orientation**	598			248			131			105			114			< 0.001
Homosexual/gay		230	(38.5%)		70	(28.2%)		54	(41.2%)		41	(39.1%)		65	(57.0%)	
Heterosexual		13	(2.2%)		2	(0.8%)		1	(0.8%)		3	(2.9%)		7	(6.1%)	
Transsexual/transgender		19	(3.2%)		15	(6.1%)		1	(0.8%)		1	(0.9%)		2	(1.7%)	
Bisexual		336	(56.2%)		161	(64.9%)		75	(53.2%)		60	(57.1%)		40	(35.1%)	
**Self-identified gender**	617			248			133			121			115			< 0.001
A man/a boy		354	(57.4%)		147	(59.3%)		61	(45.9%)		63	(52.1%)		83	(72.2%)	
Both a man and a woman		179	(29.0%)		67	(27.0%)		45	(33.8%)		43	(35.5%)		24	(20.9%)	
Much more a woman		75	(12.1%)		32	(12.9%)		27	(20.3%)		8	(6.6%)		8	(7.0%)	
Neither man nor woman		9	(1.5%)		2	(0.8)		0	–		7	(5.8%)		0	–	
**Sexual attraction**	617			248			133			121			115			< 0.001
To men		319	(51.8%)		92	(37.1%)		86	(64.7%)		60	(49.6%)		81	(70.4%)	
To men and women		269	(43.6%)		151	(60.9%)		40	(30.1%)		53	(43.8%)		25	(21.7%)	
To women		29	(4.7%)		5	(2.0%)		7	(5.3%)		8	(6.6%)		9	(7.87%)	
**Condom use during insertive anal sex**^b^	610			245			130			121			114			< 0.001
Consistent		190	(31.2%)		72	(29.4%)		36	(27.7%)		46	(38.0%)		36	(31.5%)	
Inconsistent		185	(30.3%)		55	(22.4%)		42	(32.3%)		47	(38.9%)		41	(36.0%)	
No insertive anal sex		237	(38.5%)		118	(48.2%)		52	(40.0%)		28	(23.1%)		37	(32.5%)	
**Condom use during receptive anal sex**^b^	611			246			130			121			114			0.278
Consistent		168	(27.5%)		73	(29.7%)		29	(22.3%)		34	(28.1%)		32	(28.1%)	
Inconsistent		206	(33.7%)		79	(32.1%)		56	(43.1%)		35	(28.9%)		36	(31.6%)	
No receptive anal sex		237	(38.8%)		94	(38.2%)		45	(35.6%)		52	(43.0%)		46	(40.3%)	
**Received payment (whether financial or other) for transactional sex with male partners**^b^	611			246			130			121			114			0.144
Never		417	(68.2%)		157	(63.8%)		94	(72.3%)		90	(74.4%)		76	(66.7%)	
Sometimes/always		194	(38.8%)		89	(36.1%)		36	(27.7%)		31	(25.6%)		38	(33.3%)	
**Provided payment (whether financial or other) for transactional sex with male partners**^b^	611			246			130			121			114			0.055
Never		540	(88.4%)		214	(87.0%)		122	(93.8%)		109	(90.1%)		96	(83.3%)	
Sometimes/always		71	(11.6%)		32	(13.0%)		8	(6.2%)		12	(9.9%)		19	(16.7%)	
**Group sex with male partners**^b^	611			246			130			121			114			< 0.001
Never		456	(74.6%)		197	(80.1%)		82	(63.1%)		82	(67.8%)		95	(83.3%)	
Once		75	(12.3%)		21	(8.5%)		20	(15.4%)		23	(19.0%)		11	(9.7%)	
Twice or more		80	(13.1%)		28	(11.4%)		28	(21.5%)		16	(13.2%)		8	(7.0%)	
**Number of male sexual partners**^b^	612			247			130			121			114			< 0.001
1		187	(30.5%)		92	(37.2%)		31	(23.8%)		28	(23.1%)		36	(31.6%)	
2–5		351	(57.3%)		130	(52.6%)		71	(54.6%)		82	(67.8%)		68	(59.7%)	
6–10		54	(8.8%)		18	(7.3%)		20	(14.4%)		9	(7.4%)		7	(6.1%)	
> 10		20	(3.3%)		7	(2.8%)		8	(6.2%)		2	(1.7%)		3	(2.6%)	
**Received psychological support**	512	382	(74.6%)	211	147	(69.7%)	113	90	(79.6%)	89	67	(75.3%)	99	78	(78.8%)	0.171
**History of HIV screening**	618	532	(86.1%)	249	218	(87.6%)	133	113	(85.0%)	121	96	(79.3%)	115	105	(91.3%)	0.054
**STI (other than HIV) symptoms**	618	78	(12.6%)	249	13	(5.2%)	133	17	(12.8%)	121	37	(30.6%)	115	11	(9.6%)	< 0.001

^a^ Median (interquartile range)

^b^ During the previous 6 months

### Adherence to quarterly follow-up visits

Overall adherence to quarterly HIV prevention follow-up visits was 76.5% (95% confidence interval [CI] 75.1–77.8) over a median observation time of 20.0 months (IQR 15.2–26.3). Fifty-one participants (8.3%) never attended the clinics after the enrolment visit, and an additional 52 (8.4%) did not attend at least their last two scheduled follow-up visits. Two other participants died from unknown reasons. Specifically, adherence to quarterly follow-up visits was 87.3% (95% CI 84.0–90.1) over a median observation time of 15.8 months (IQR 14.7–16.6) in Lomé, 78.9% (95% CI 77.0–80.7) over 26.9 months (IQR 21.4–29.3) in Bamako, 73.2% (95% CI 69.4–76.5) over 17.3 months (IQR 14.0–19.5) in Ouagadougou, and 66.8% (95% CI 63.4–70.0) over 23.8 months (IQR 13.1–25.8) in Abidjan.

**Fig. 1:**
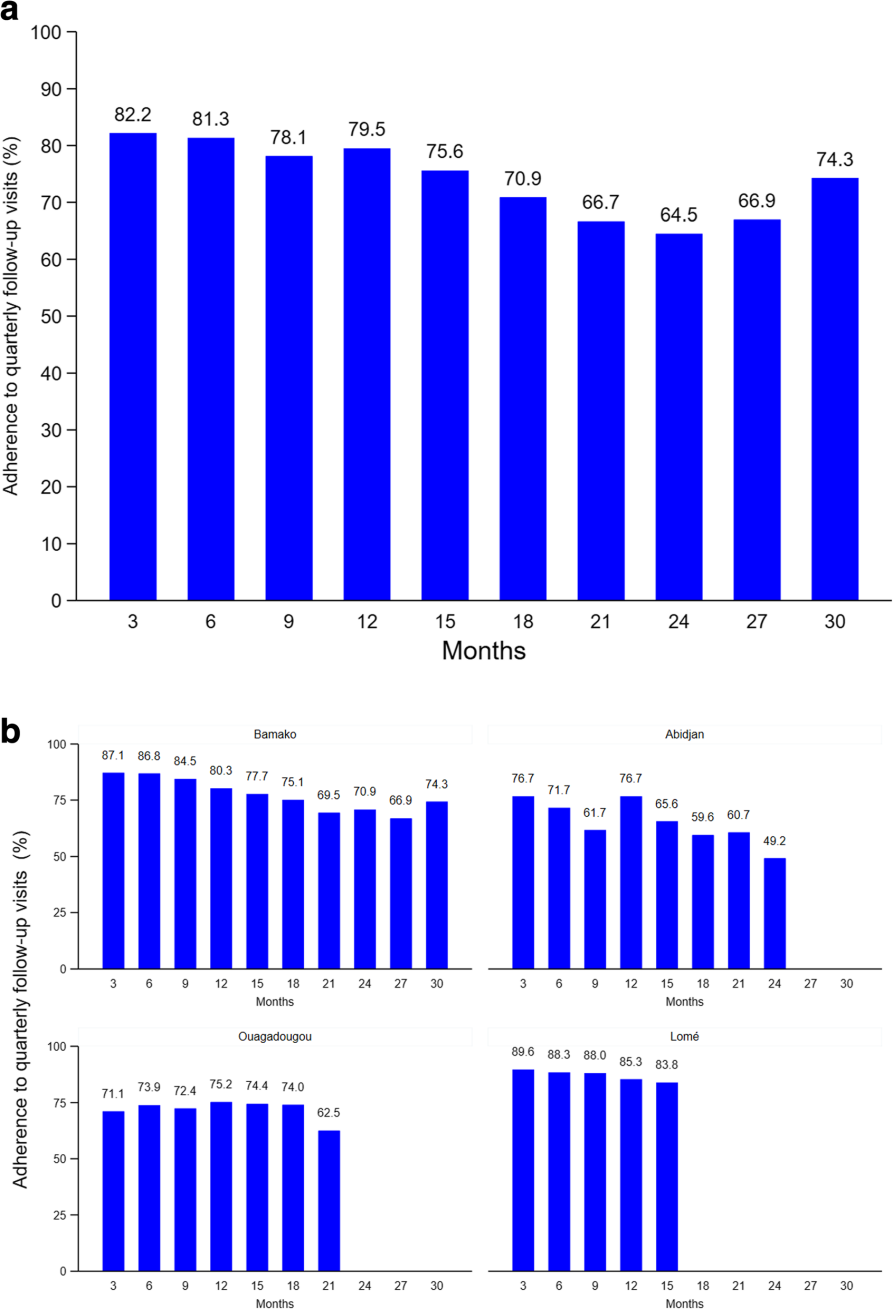
Adherence to quarterly follow-up visits (**a**) in all study cities (**b**) according to study city

Adherence to quarterly follow-up visits was highest at month 3 (82.2%) and lowest at month 24 (64.5%; Fig. 1). Overall, it decreased significantly over time (*p* < 0.001). The decrease was significant in Bamako and Abidjan (*p* < 0.001 for both) but not in Ouagadougou (*p* = 0.887) and Lomé (*p* = 0.730).

In multivariate analysis including observation time, adherence to quarterly follow-up visits was significantly higher in Bamako (adjusted coefficient [aβ] 0.17, 95% CI 0.11; 0.22, *p* < 0.001) and Lomé (aβ 0.15, 95% CI 0.08; 0.21, *p* < 0.001), but not in Ouagadougou (aβ 0.05, 95% CI − 0.01; 0.12, *p* = 0.107), than in Abidjan (Table 2). Multivariate analysis also confirmed that adherence decreased with time (aβ − 0.01 per 1-month increase, 95% CI -0.02; − 0.01, *p* < 0.001). Adherence was not associated with any other participant characteristic.

**Table 2:** Determinants of adherence to quarterly follow-up visits (generalized estimating equations regressions)

	Univariate analysis			Multivariate analysis		
	β	95% CI	*P*	aβ	95% CI	*P*
**City**						
Abidjan	Reference			Reference		
Bamako	0.13	0.10; 0.17	< 0.001	0.17	0.11; 0.22	< 0.001
Ouagadougou	0.10	0.06; 0.14	< 0.001	0.05	−0.01; 0.12	0.107
Lomé	0.21	0.17; 0.26	< 0.001	0.15	0.08; 0.21	< 0.001
**Observation time (per 1-month increase)**	− 0.01	− 0.01; − 0.007	< 0.001	-0.01	−0.02; − 0.01	< 0.001
**Age (years)**						
≤ 25	Reference					
> 25	−0.01	− 0.04; 0.01	0.288			
**Educational level**						
No school/Koranic	Reference					
Primary/secondary/university	−0.03	−0.07; 0.02	0.222			
**Marital status**						
Single/divorced/separated/widowed	Reference					
Married/free union	−0.02	− 0.04; 0.004	0.109			
**Self-defined sexual orientation**						
Transsexual/transgender	Reference					
Homosexual or gay/heterosexual/bisexual	0.009	−0.03; 0.04	0.620			
**Self-identified gender**						
A man/a boy	Reference					
Other	−0.001	−0.02; 0.01	0.853			
**Sexual attraction**						
To men	Reference					
To men and women/to women	0.0004	−0.01; 0.01	0.945			
**Condom use during insertive anal sex**^a^						
Consistent	Reference					
Inconsistent	−0.003	− 0.02; 0.01	0.694			
No insertive anal sex	−0.002	−0.02; 0.01	0.765			
**Condom use during receptive anal sex**^a^						
Consistent	Reference					
Inconsistent	−0.004	−0.02; 0.01	0.615			
No receptive anal sex	−0.004	−0.02; 0.01	0.560			
**Received payment (whether financial or other) for transactional sex with male partners**^a^						
Never	Reference					
Sometimes	0.002	−0.01; 0.01	0.760			
Always	−0.002	−0.03; 0.03	0.894			
**Provided payment (whether financial or other) for transactional sex with male partners**^a^						
Never	Reference					
Sometimes	−0.0005	−0.02; 0.02	0.955			
Always	−0.009	−0.06; 0.08	0.804			
**Group sex with male partners**						
Never	Reference					
Once	−0.01	−0.04; 0.01	0.170			
Twice or more	−0.02	− 0.05; 0.01	0.109			
**Number of male sexual partners**^a^						
1–5	Reference					
≥ 6	−0.0002	−0.01; 0.11	0.968			
**Received psychological support**						
No	Reference					
Yes	0.007	−0.01; 0.22	0.353			
**History of HIV screening**						
Yes	Reference					
No	0.05	0.01; 0.09	0.018			
**STI (other than HIV) symptoms**						
No	Reference					
Yes	−0.004	−0.02; 0.02	0.712			

*Abbreviations*: *CI* confidence interval, *STI* sexually transmitted infection

^a^During the previous 6 months

### HIV incidence

**Fig. 2:**
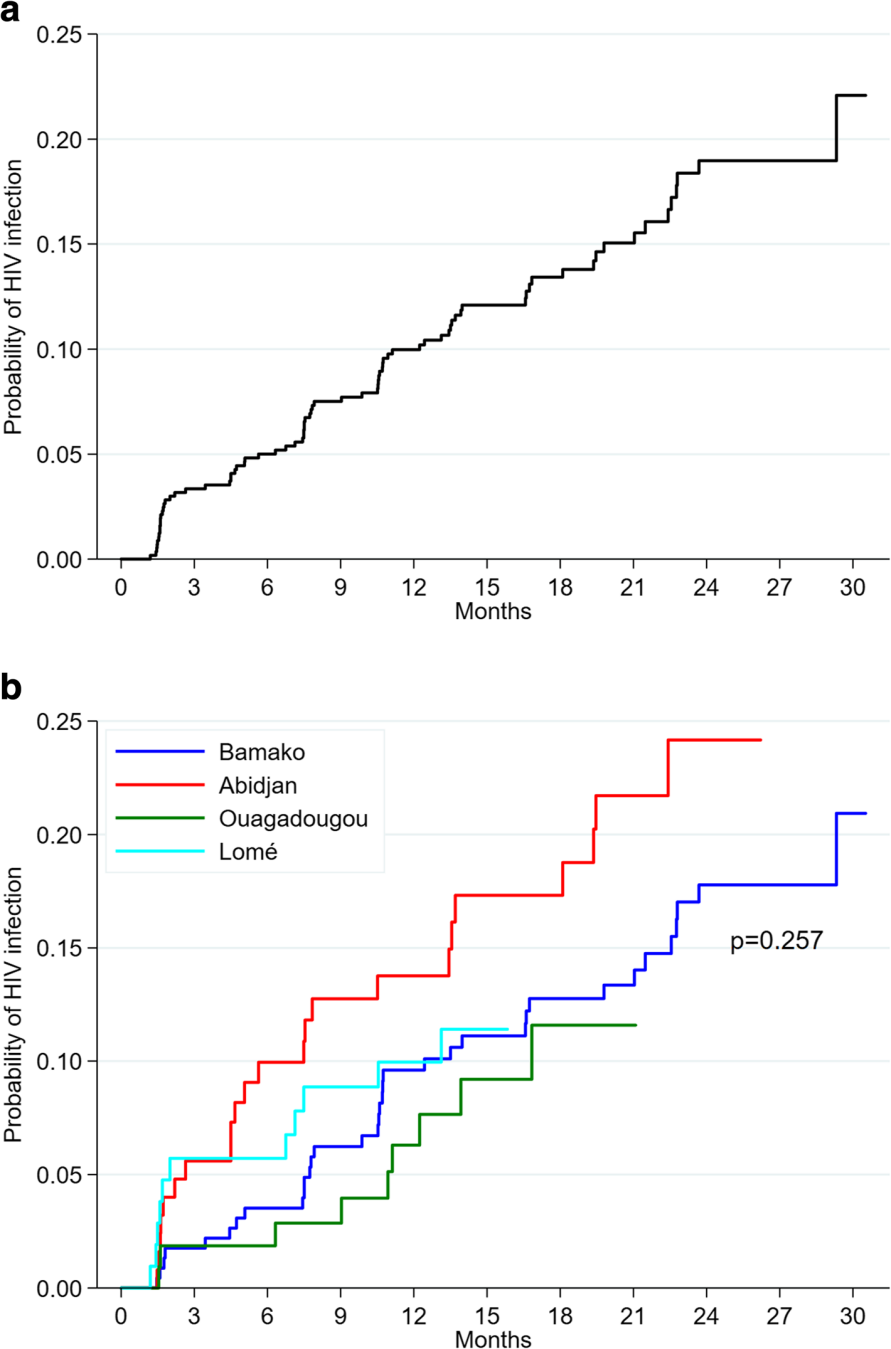
Cumulative probability of incident HIV infections (**a**) in all study cities (**b**) according to study city. *P*-value calculated using the log-rank test

Seventy-eight participants seroconverted over a total follow-up time of 780.8 person-years, giving an overall HIV incidence of 10.0 per 100 person-years (95% CI 8.0–12.5). Incident HIV infections occurred after a median time of 7.8 months (IQR 3.4–13.5). HIV incidence was 14.4 per 100 person-years (95% CI 9.6–21.7) in Abidjan, 10.2 per 100 person-years (95% CI 5.7–18.5) in Lomé, 9.0 per 100 person-years (95% CI 6.5–12.5) in Bamako, and 7.3 per 100 person-years (95% CI 3.8–14.0) in Ouagadougou. The time of incident HIV infections did not differ significantly between the study cities (*p* = 0.257; Fig. 2).

Table 3 shows HIV incidence according to participant characteristics. The risk of incident HIV infection was not significantly associated with adherence to quarterly follow-up visits in either univariate (hazard ratio [HR] 0.69, 95% CI 0.39–1.23, *p* = 0.209) or multivariate analysis (adjusted hazard ratio [aHR] 0.80, 95% CI 0.44–1.44, *p* = 0.545). However, it was significantly higher in participants who did not consistently use condoms during insertive anal sex (aHR 3.09, 95% CI 1.52–6.32, *p* = 0.002) and those who reported no insertive anal sex (aHR 3.77, 95% CI 1.90–7.50, *p* < 0.001) in the previous six months than in participants who consistently used condoms during insertive anal sex. Finally, it was significantly higher in participants who had never been tested for HIV before study enrolment than in those who had been (aHR 2.48, 95% CI 1.41–4.36, *p* = 0.002).

**Table 3:** HIV incidence and determinants of incident HIV infections (Cox models)

	Incident HIV cases/person-years	Incidence/100 person-years (95% CI)		Univariate analysis			Multivariate analysis		
				HR	95% CI	*P*	aHR	95% CI	*P*
Adherence to quarterly follow-up visits (%)		11.6	(7.1–19.0)	1			1		
< 100	16/137.4			0.69	0.39–1.23	0.209	0.80	0.44–1.44	0.545
100	62/643.4	9.6	(7.5–12.4)						
**City**									
Abidjan	23/159.8	14.4	(9.6–21.7)	1			1		
Bamako	35/389.7	9.0	(6.4–12.5)	0.61	0.36–1.04	0.070	0.67	0.39–1.16	0.156
Ouagadougou	9/123.7	7.3	(3.8–14.0)	0.48	0.22–1.05	0.066	0.49	0.22–1.07	0.073
Lomé	11/107.6	10.2	(5.6–18.5)	0.66	0.31–1.36	0.256	0.90	0.42–1.91	0.783
**Age (years)**									
≤ 25	50/490.4	10.2	(7.7–13.4)	1					
> 25	28/290.4	9.6	(6.6–14.0)	0.94	0.59–1.50	0.808			
**Educational level**									
No school/Koranic	2/30.4	6.6	(1.6–26.3)	1					
Primary/secondary/university	68/727.5	9.3	(7.4–11.8)	1.43	0.35–1.03	0.615			
**Marital status**									
Single/divorced/separated/widowed	50/616.1	8.1	(6.1–10.7)	1					
Married/free union	20/141.8	14.1	(9.1–21.9)	1.78	1.06–2.99	0.030			
**Self-defined sexual orientation**									
Transsexual/transgender	4/17.0	23.6	(8.8–62.7)	1					
Homosexual or gay/heterosexual/bisexual	67/740.2	9.1	(7.1–11.5)	0.41	0.15–1.13	0.084			
**Self-identified gender**									
A man/a boy	39/475.8	8.2	(6.0–11.2)	1					
Other	34/289.7	11.7	(8.4–16.4)	1.44	0.91–2.28	0.123			
**Sexual attraction**									
To men	33/407.1	8.1	(5.8–11.4)	1					
To men and women/to women	40/366.1	10.9	(8.0–14.9)	1.34	0.84–2.08	0.214			
**Condom use during insertive anal sex**^a^									
Consistent	11/294.3	3.7	(2.1–6.4)	1			1		
Inconsistent	27/232.8	11.6	(7.9–16.9)	2.97	2.14–13.31	0.003	3.09	1.51–6.32	0.002
No insertive anal sex	34/242.9	14.0	(10.0–19.6)	3.72	1.97–11.22	< 0.001	3.77	1.90–7.50	< 0.001
**Condom use during receptive anal sex**^a^									
Consistent	25/222.8	11. 2	(7.6–16.6)	1					
Inconsistent	29/207.0	14.0	(9.7–20.2)	1.21	0.91–3.00	0.494			
No receptive anal sex	18/340.0	5.3	(3.3–8.4)	0.46	0.23–0.86	0.013			
**Received payment (whether financial or other) for transactional sex with male partners**^a^									
Never	53/550.3	9.6	(7.4–12.6)	1					
Sometimes	16/182.5	8.8	(5.4–14.3)	0.91	0.52–1.60	0.749			
Always	3/20.9	14.4	(4.6–44.6)	1.49	0.46–4.77	0.509			
**Provided payment (whether financial or other) for transactional sex with male partners**^a^									
Never	67/679.2	9.9	(7.8–12.5)	1					
Sometimes	5/72.0	6.9	(2.9–16.7)	0.70	0.28–1.74	0.442			
Always	0/2.5	–		NC	–	–			
**Group sex with male partners**^a^									
Never	62/681.0	9.1	(7.1–11.7)	1					
Once	6/40.9	14.7	(6.6–32.7)	1.23	0.43–3.47	0.697			
Twice or more	4/31.8	12.6	(4.7–33.5)	1.53	0.65–3.61	0.331			
**Number of male sexual partners**^a^									
1–5	63/687.0	9.2	(7.2–11.7)	1					
≥ 6	10/88.0	11.4	(6.1–21.1)	1.26	0.65–2.47	0.494			
**Received psychological support**									
No	9/120.1	7.4	(3.9–14.3)	1					
Yes	55/608.7	9.0	(6.9–11.8)	1.22	0.60–2.49	0.580			
**History of HIV screening**									
Yes	62/695.0	8.9	(7.0–11.4)	1			1		
No	16/85.7	18.7	(11.4–30.5)	2.05	1.18–3.57	0.011	2.48	1.41–4.36	0.002
**STI (other than HIV) symptoms**									
No	69/729.4	9.4	(7.5–12.0)	1					
Yes	5/50.8	9.8	(4.1–23.7)	1.04	0.42–2.58	0.935			

*Abbreviations*: *HR* hazard ratio, *aHR* adjusted hazard ratio, *CI* confidence interval, *NC* not calculable, *STI* sexually transmitted infection

^a^During the previous 6 months

## Discussion

This multi-country study conducted in MSM living in West Africa showed good overall adherence to quarterly HIV prevention services, which included HIV testing and counselling, screening and treatment for other STI, and the provision of condoms and lubricants. However, we recorded a decrease in adherence over time as well as differences between the study cities (Abidjan, Bamako, Lomé, and Ouagadougou). Moreover, adherence had no significant impact on HIV incidence, which was high in the study population.

The good overall adherence to quarterly follow-up visits by HIV-negative individuals is encouraging for the implementation of prevention programmes, as MSM commitment is crucial for repeated HIV testing and counselling, screening and treatment for other STI, as well as PrEP (not examined here). Importantly, repeated HIV testing was well accepted by participants and was performed systematically at each visit. The good adherence observed in the study was likely related to the favourable study context, specifically the fact that the study clinics were MSM-friendly, and that peer-educators were very involved in enrolment and retention of MSM in the programme, as well as counselling and psychosocial support [23]. Adherence support for MSM included a reminder telephone call 15 days after an outstanding follow-up visit. This measure was inspired by routine practice in the study clinics, in which medical teams call HIV-positive patients if they are late for scheduled visits. Mobile phones are now widely used throughout Sub-Saharan Africa, and just as is the case for ART, they constitute a useful and affordable tool for maintaining adherence to prevention services. The compensation of US$5 for transport costs for each follow-up visit certainly encouraged adherence. Although the use of financial compensation for routine medical interventions has been debated, its effectiveness has been shown in different contexts [24, 25].

However, two of our findings on adherence to quarterly HIV prevention services call for caution. First, despite having the same study procedures, lower adherence was observed in Abidjan than in Bamako and Lomé, which suggests that the local context (e.g., difficulty and cost of transportation, reluctance/willingness to come to clinics, and spatial and temporal organization of clinics) had an impact. This underlines the need for additional measures adapted to the local context in terms of adherence support for MSM and solutions to overcome organizational constraints in clinics. Second, the observed decrease of adherence over time could be problematic for long-term public health programmes. However, the relatively small proportion of participants lost to follow-up shows that most of the missed visits were accounted for MSM still in the programme. We hypothesize that long-term adherence to follow-up will be better in MSM using PrEP who will need to come to medical visits for supplies.

Despite the good adherence to quarterly prevention services, HIV incidence in our study was far higher than the WHO-recommended threshold of 3 per 100 person-years, which defines populations at substantial risk and who should be offered PrEP [26]. Our figure was comparable with those from other African studies in which incidence was 6.8 per 100 person-years (95% CI 4.9–9.2) and 8.6 per 100 person-years (95% CI 6.7–11.0) in two different studies in Kenya, and 16.0 per 100 person-years (95% CI 4.6–27.4) and 15.4 per 100 person-years (95% CI 12.3–19.0) in studies in Senegal and Nigeria, respectively [13, 27–29]. Although HIV incidence did not differ statistically between the four study cities, our data confirm that it was especially high in Abidjan, reflecting findings in CohMSM’s pilot study in 2013–2015 (15.9 per 100 person-years, 95% CI 7.6–33.4) [30]. In a previous analysis, we found that MSM at greater risk of exposure to HIV infection decreased their risky sexual behaviours during their follow-up in CohMSM, suggesting a potential positive effect of the quarterly prevention services on those who need it most [31]. However, the high HIV incidence and the lack of association between adherence to these services and incident HIV infections observed in the present analysis strongly suggest that this intervention alone is insufficient to significantly reduce the burden of the epidemic in this population. This may be due to the fact that most HIV infections occurred in the first months of follow-up while changes in sexual behaviours require more time and are rarely optimal. These data confirm the need to strengthen HIV prevention services through the use of PrEP.

In our study, some participants did not attend the quarterly follow-up visits because they had moved elsewhere, either temporarily or permanently. Only when participants moved from one study city to another - which was quite common in the study population - could they continue their follow-up in the second study clinic. This underscores the need to establish a network of intra-country and inter-country MSM-friendly clinics in West Africa (and elsewhere) for sustainable prevention.

A major challenge for the control of HIV infection in MSM is to reach and test those who have never been tested. In our study, these men were at greater risk of acquiring HIV infection. A recent study in neighbouring Nigeria reported a similar finding [13]. The relationship between condom use during insertive anal sex and incident HIV infections reflects existing evidence, as inconsistent condom use is a well-known risk factor and most participants who reported no insertive anal sex also reported receptive anal sex.

The main strength of this study is the fact that it was conducted in four West African countries, which allowed us to highlight differences in the outcomes between all four. However, our findings have the following limitations. First, our participants might not be fully representative of the global MSM community in the four study cities because the study was performed in MSM enrolled and followed up in MSM-friendly community-based clinics. Second, the study was performed in the major cities of Burkina Faso, Côte d’Ivoire, Mali, and Togo and our results may not be generalizable to other contexts of these countries. Third, the duration of the study differed between countries due to the staggered start of the study and the staggered discontinuation of the study. Finally, a social desirability bias may have affected participants’ responses (e.g., regarding condom use).

## Conclusions

This multi-country study clearly demonstrates that strengthening HIV prevention services among MSM in West Africa will be critical for controlling the epidemic, not only in this key population but also in the general population. Fortunately, West African countries are currently reviewing their national guidelines and are planning to integrate PrEP into their prevention services. This study’s results suggest that quarterly follow-up of MSM, which is essential for PrEP delivery, is feasible.

## Data Availability

Due to French law there are restrictions on publicly sharing the data of this study. French law requires that everyone who wishes to access cohort data or clinical study data on humans must make a request to the French Data Protection Authority (Commission Nationale de l’Informatique et des Libertés - CNIL), by filling in a form which can be provided by Christian Laurent at the IRD (christian.laurent@ird.fr). For further information, please see: https://www.cnil.fr/.

## Abbreviations

aHR: Adjusted hazard ratio
CI: Confidence interval
HIV: Human immunodeficiency virus
HR: Hazard ratio
IQR: Interquartile range
MSM: Men who have sex with men
PrEP: Pre-exposure prophylaxis
STI: Sexually transmitted infection
WHO: World Health Organization
